# Interventions for Preventing Childhood Obesity with Smartphones and Wearable Device: A Protocol for a Non-Randomized Controlled Trial

**DOI:** 10.3390/ijerph14020184

**Published:** 2017-02-13

**Authors:** Hye Jung Yang, Jae-Heon Kang, Ok Hyun Kim, Mona Choi, Myungju Oh, Jihyun Nam, Eunju Sung

**Affiliations:** 1 Department of Family Medicine, Kangbuk Samsung Hospital, Sungkyunkwan University School of Medicine, Seoul 03181, Korea; katie.in.seoul@gmail.com (H.J.Y.); likeu7@naver.com (J.N.); 2 Department of Family Medicine, Seoul Paik Hospital, College of Medicine, Inje University, Seoul 04551, Korea; fmleader@nuri.net; 3 Institute for Clinical Nutrition, Inje University, Seoul 04551, Korea; okhyunee@hanmail.net; 4 Nursing Research Institute, College of Nursing, Yonsei University, Seoul 03722, Korea; monachoi@yuhs.ac; 5 Department of Family Medicine, Dae-Chung Hospital, Daejeon 35403, Korea; dhaudwn79@naver.com

**Keywords:** childhood obesity, prevention, smartphone, early intervention

## Abstract

*Background*: Childhood obesity is a critical health issue, both currently and for the foreseeable future. To prevent obesity, behavior changes are essential. Smartphones can be a good tool, as the number of child smartphone users is rapidly increasing. We have developed a mobile platform system named “HAPPY ME,” which is a smartphone application coupled with a wearable device, designed to improve healthy behaviors to prevent childhood obesity. This study aimed to evaluate the effectiveness of obesity prevention among children 10–12 years of age using HAPPY ME. *Methods*: A total of 1000 participants, all fifth and sixth graders from four schools, were assigned to either control or intervention groups by school. Students in the intervention group used HAPPY ME. The study comprises a safety test, a 12-week efficacy test, and a six-month follow-up test to determine the long-term effects of preventive intervention via the integrated service platform. The integrated service platform aims to facilitate child-parent-school participation, involving the child-parent mobile application, a child-teacher mobile web, and a school website. Primary outcome measures are behavioral changes, including healthy eating, increased physical activity, and fitness. Secondary outcome measures are changes in anthropometric parameters (body weight, height, body mass index z-score, and waist circumference), body mass index (BMI) percentiles (obesity rate), and psychological perceptions among participants. *Conclusions*: The results of this study will offer evidence of the effectiveness of a mobile platform service with a multi-component intervention program based on a comprehensive approach.

## 1. Introduction

The rate of childhood obesity in Korea is constantly increasing, with 26.4% of boys categorized as either overweight or obese as of 2013, exceeding the Organization for Economic Cooperation and Development (OCED) average of 24.3% [1,2]. Showing the disparity between males and females in terms of childhood obesity [2], 14.1% of girls are categorized as being in the same condition. According to the measure of BMI for children and teens by the Centers for Disease Control and Prevention (CDC), childhood obesity is defined as a BMI at or above the 95th percentile for children of the same age and sex, whereas overweight is defined as a BMI at or above the 85th percentile and below the 95th percentile for children of the same age and sex [3]. Childhood obesity can seriously impact public health matters, not only causing enormous medical expenses, but also resulting in various illnesses over the lifetime [4,5]. For these reasons, numerous studies have focused on childhood obesity prevention and management intervention [6,7,8,9,10]. Also, research on childhood obesity has highlighted the necessity of combined dietary-behavioral-physical activity intervention along with family-based education to foster a supportive home environment, which is integrated with a child’s school system [11,12,13,14,15,16]. School-based approaches have been shown to be the most effective in solving the health problems of school-age children, including obesity [13,17,18,19]. Recent studies have introduced new technologies to help prevent or manage obesity, using smartphone applications that tackle issues of dietary and physical activity (PA), together with wearable devices that track the intensity of the user’s PA as well as daily and weekly calorie consumption [20,21,22,23,24,25,26]. Insofar as behavior changes are essential for the prevention and treatment of obesity [27], mobile health (mHealth)—a general term that refers to the use of mobile phones and other wireless technology in medical care—can be a useful tool in implementing and monitoring behavior changes [28]. Types of intervention that involve smartphone-assisted programs have been shown to be effective in obese adolescents [29]. Evidence is lacking, however, as to whether these types of intervention programs are well suited for school-age children. Regardless, mobile applications targeting children to promote healthier behaviors have potential, because many youth begin interacting with computers and cell phones at very early ages [30]. Thus, smartphones, if used in childhood obesity intervention programs, will be familiar tools for children. According to the Korea Information Society Development Institute as of 2015, 72.3% of upper-grade elementary school students have their own cellular phones. Among these, 59.3% have smartphones [31]. In the findings of our first pilot study, smartphones were owned by 55.2% (boys, 49.5% and girls, 60.8%) of school-age children in grades four through six. Accordingly, we assumed that our approach to the problem of childhood obesity with smartphones is plausible. It has been stated in previous studies that large-scale data for childhood obesity prevention with smartphones are lacking [12,32]. This trial will be the first to assess childhood obesity prevention in a school setting (also including parents) with a multi-component program using a mobile platform system called HAPPY ME, operated via a smartphone application and a wearable device. Our goal with this ongoing trial is to evaluate the effectiveness of childhood obesity prevention with the HAPPY ME platform for students, parents, and teachers.

## 2. Methods

### 2.1. Study Participants

**Figure 1:**
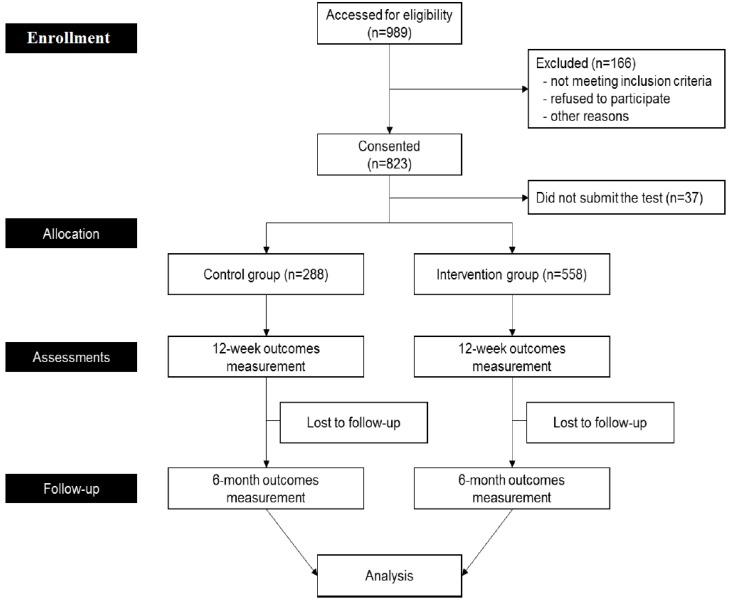
Flow chart of the study.

Participating schools were recruited by a sanctioned announcement involving official letters from the Metropolitan and Provincial Offices of Education in Gyeonggi Province. Since the recruitment publicity included information regarding the use of mHealth to tackle childhood obesity, the study could not be masked and all applicant schools wished to be designated as intervention schools. We took pragmatic approach to intervention and control group allocation due to the fact schools were unwilling to be randomized. According to our pilot study conducted the previous year, less than 40% of students in fourth grade have smartphones, which can lead to a serious selection bias. The pilot study showed that the number of students who own smartphones increases as students advance through grades at school. Therefore, we determined that fifth and sixth graders were eligible to participate in our research by following a nine-month-long study using smartphones and wearable devices for the prevention of childhood obesity. The sample size was determined based on the government funding, the feasibility of the study, the number of measurements per participant, the number of students we could recruit, and the potential dropout rate considering the age of participants rather than the effect sizes of the intervention. We chose 250 students from each school as an appropriate number, for a total of 1000 students across four participating schools. To categorize children’s weight status, we used the standard growth chart for infants and children by the Korea Centers for Disease Control and Prevention [33]. Written consent was collected from all participating children and their parents. Inclusion criteria were fifth-sixth graders (10–12 years old), no previous diagnosis of systemic or metabolic diseases, and Android smartphone users with parental consent, students who did not refuse to participate (see Figure 1). Finally, 288 students were assigned to a control group, and 558 students were allocated in the intervention group. Any physical restrictions in participants were assessed during meetings with teachers and in loading the program to the smartphone applications of participants.

#### 2.1.1. Control Group

Students in the control group underwent the same measurements as students in the intervention group, including Physical Activity Promotion System (PAPS) measurements and lifestyle questionnaires administered at the baseline, at week 12, and at month 6. Comprehensive assessments of dietary habits, physical activities, and psychological variables are planned for each assessment period using questionnaires. The control group received the same two group education sessions. Data for these 250 students from one elementary school were collected.

#### 2.1.2. Intervention Group

##### HAPPY ME Platform

In this trial, we developed the HAPPY ME platform, which involves parents and teachers in monitoring and providing an encouraging environment for participating children. The platform serves as a data reservoir for teachers, enabling them to track students’ eating behaviors, screen time, and anthropometric parameters. Data are shown in numbers and graphs and allow teachers to support the children with encouraging messages. In addition, educational materials on healthy eating habits and exercise are included in the platform content. This information is featured as guidelines for teachers and parents. The platform delivers feedback messages to children and parents. The application logic for the selection of feedback for the HAPPY ME platform for parents and children differs according to the differing needs of participants in this study. The platform sends PAPS data and performance data from the applications of students to a mobile web for teachers.

##### Intervention for Children

Among 750 students from the three intervention schools, students who met inclusion criteria for this study downloaded the “HAPPY ME for students” application. Due to financial and time limits of the developer, the application was developed for Android systems only. This application serves as a self-monitoring tool that focuses on factors known to be associated with childhood obesity including dietary habits, screen time, sleep duration (measured in hours), and physical activity [16,24,25,34,35,36,37,38]. As previously described, HAPPY ME facilitates healthy behavior decisions using gamification and self-monitoring strategies [38,39]. The application sends tailored messages to children to encourage them to complete daily quests for physical activities and to practice healthy dietary habits, such as eating breakfast, choosing fruits and vegetables, and avoiding sugar-sweetened beverages and fast foods, all of which are helpful habits in preventing childhood obesity. The application collects information about participants’ dietary habits and physical performance to categorize and offer optimal feedback to achieve behavioral changes to prevent obesity. Herein, the participating students recorded basic information including weight, height, dietary behavior, and number of hours of sleep at the beginning of the application. Students used height information measured by PAPS at the beginning of the study. Input data in the application automatically categorized the individual status of each student into six categories of dietary behavior and three categories of fitness within the application. For dietary behavior, we defined the following six categories as standard and optimal goals for healthy diets in children: consumption of vegetables five times per day, consumption of fruits twice a day, drinking water instead of sugar-sweetened beverages (SSBs), eating a healthy meal instead of fast or instant food, not eating snacks, and not eating late at night. For physical performance, we defined the following three categories to measure the proper amount of walking per day according to sex: high activity, moderate activity, and low activity. The high activity category includes activities with a number of counted steps over 14,000 steps for boys and over 12,000 steps for girls. Moderate activities are defined as 7000–14,000 steps for boys and 6000–12,000 steps for girls, and low activities are measured as under 7000 steps for boys and under 6000 steps for girls [40]. Once the individual is categorized according to behavior, the application presents a dietary and physical activity quest to encourage users to practice better health behaviors to prevent obesity. Students choose one challenge among the quests presented, and the mission lasts for one week. A tailored message is offered once a day to users based on their performance of the selected quest. After one week, children may choose another quest or continue the previous quest if the quest has not been successfully completed. Eventually, students complete all of the quests according to the guidelines of the application. Participants can monitor their daily and weekly performances, and the application provides feedback and socio-emotionally supportive messages according to user behaviors. These messages are generated from a message pool that the research team developed under a trans-theoretical model. Messages are automatically sent to students according to the logics of each performance. To add a gamification element to the application, children get points if they have completed daily quests, which in turn leads to a higher level of fitness and a healthier diet. Teachers and parents are able to assess how well individuals are doing by comparing these scores among classmates through the application as a series of gamifying elements and, accordingly, to better motivate students to complete the quests assigned to them. If the children do not wish to compare the scores with classmates, they have an option in the application not to be compared. Participants in the intervention group also received an activity tracker called the Walkie + D Coffee (Green Cross Healthcare Inc., Seoul, Korea) via the research team at each school. According to tracker accuracy tests conducted by our developers, the accuracy between volume of oxygen uptake (VO2) and calorie expenditure (kcal/min) through gas analysis and the expenditures of calories according to step count by the device was 80% [41]. Therefore, we considered this device to be a usable activity tracker. Students were instructed to wear the device on their wrist. Daily, weekly, and monthly physical activity data, such as intensity of workouts, step count, and number of calories expended by walking or running with the Walkie + D Coffee, are transferred via Bluetooth to the participant’s smartphone.

##### Intervention for Parents

Parents downloaded the “HAPPY ME for parents” application to receive feedback from the platform and to monitor their child’s dietary habits and physical activity. The main role of parents in this study is to provide their children with an optimal environment to improve healthy behaviors. Educational materials about obesity, such as risks of obesity, healthy eating habits, and nutrition, are included for reference if necessary. As the main food preparers and emotional supporters of the participants, parents receive tailored messages once a week in the context of the child’s performance to encourage them on their weekly missions. The tailored messages for parents also offer guidance on how to create a healthier home environment.

##### Intervention for Teachers

The main role of teachers in this prevention-based intervention program is to encourage students to improve healthy behaviors rather than to monitor participants’ performance (which is optional). The scope and content for the teacher websites at the schools comprising the intervention group (“HAPPY ME for teachers”) were created according to teacher opinions based on focus-group interviews and a need assessment survey. The primary teacher opinion was to minimize extra work on the part of teachers for this program to guarantee feasibility of the program in the real school system. Teachers are very busy with their original duties. It is important to reduce the burden on teachers and to support their original educational duties. We asked teachers in charge of classes with participating students to encourage healthy behaviors among their students at least once a week. The website is open for all teachers at participating schools. The content of the website comprises teacher-friendly educational material about nutrition and physical activity, test results of physical fitness measures among participants, and monitoring capabilities regarding application data on participating students from their classes. The educational materials contain information to enhance the performance of key quests in the application. Teachers may use the educational materials during lessons to better help students in following the HAPPY ME quests during the 12-week efficacy test period. The materials provided on the teacher websites are modified to be more appropriate for teaching a health-related curriculum. Teachers in charge of classes with participating students can encourage participants (or the whole class) using a bulletin or sending messages via the website, which can then be shown in an individual’s smartphone application.

### 2.2. Study Design

The current study is a school-based nonrandomized controlled trial funded by a national grant to prevent childhood obesity in Korea, which has been undertaken utilizing a safety test, a 12-week efficacy test, and a planned subsequent six-month follow-up test for long-term effects. During the study period of 37 weeks, participating students are expected to use their smartphones along with a wearable device, while parents and teachers will receive feedback messages about the performance of participants via smartphones and teacher websites. Data is collected on the part of the research team by monitoring the HAPPY ME platform and spot feedback from teachers and children. For the feedback session with children, our research team will be assigned to each school for three months. A group meeting with parents and teachers for feedback is planned to occur during the three-month efficacy trial. To evaluate the students’ physical performance, data collected by the Physical Activity Promotion System (PAPS) from each school will be monitored. Reporting PAPS data is mandatory in the Korean school system. The PAPS consists of tests to assess cardiac endurance, muscle strength, power, flexibility, and body composition. The PAPS was developed by the Korean government according to public interest in health concerning the growing number of obese and/or weak children. Every elementary school performs PAPS testing once or twice a year to measure student health and, based on results, offers individual exercise prescriptions to every participant. For the schools participating in the application efficacy trial herein, participating students will perform PAPS testing before and after the trial under the guidance of experienced examiners to test the participants’ overall physical fitness, including obesity evaluation, by measuring percent body fat using bioelectrical impedance analysis (BIA). A lifestyle questionnaire which was designed and internally validated was conducted at the beginning of the trial. The questionnaire will also be conducted at week 12 and at the end of the trial. The questionnaire consists of behavioral factors regarding sleep, stress, dietary habits, and activity habits, as well as the amount of screen time by participating children and psychological factors, such as body image. Every participant is required to attend two group education sessions at their respective schools featuring information on a healthy diet and food additives, as well as easy-to-learn physical activities from professionals at week 0 and at week 8 during the 12-week efficacy test period (see Table 1).

#### 2.2.1. Phase 1: Safety Test

Participants in the intervention group downloaded an application called What’s UBhind? (Rinasoft Inc., Busan, Korea) to test the safety of the intervention program one week before baseline. The application provides daily, weekly, and monthly reviews of the amount of screen time involving a user’s smartphone, thereby enabling the user to accurately depict his or her smartphone screen time (ST). It is known that excessive ST is related to lower self-esteem, depression, and increased odds of obesity or being overweight. Accordingly, monitoring screen time is crucial to some obesity prevention intervention studies [16,24]. To assess ST, individuals can customize limits in the application, which will alert users as soon as the set screen time has been exceeded. Screen time is reported to the HAPPY ME websites of teachers. According to an algorithm we have developed, feedback messages on excessive screen time will be sent to both children and parents. Data as to whether this intervention involving smartphones impacts the rising amount of screen time among children will be collected and monitored by the research team. The safety test will continue until the six-month follow-up period is finished, but it is not mandatory.

#### 2.2.2. Phase 2: Efficacy Test

Participants assigned to the intervention group downloaded an application called HAPPY ME. Consenting parents and children in the intervention group participated in a workshop before baseline at each school. This workshop was led by the research team to explain the study protocol, as well as the use of the HAPPY ME application and the wearable device. During the 12-week trial, students use a wearable device called the Walkie + D Coffee. Physical activity data collected by the wearable device are transmitted directly to the HAPPY ME application. Use of the application helps children self-monitor their behavior in terms of physical activity and dietary habits. Students participated in two group education sessions at weeks 4 and 8, both of which focused on a healthy diet and physical activity. Physical Activity Promotion System (PAPS) testing and lifestyle questionnaires were conducted at the baseline and are planned for the final week of the trial.

#### 2.2.3. Phase 3: Long-Term Effect Test

Following the 12-week efficacy test, the intervention group will voluntarily continue using the application and the wearable device during a six-month follow-up period to test the long-term effects of the intervention in terms of preventing obesity. After the follow-up period, the children’s application access log and performance data will be collected by the research team. At month 6, both the intervention group and the control group will complete lifestyle questionnaires and assessment via PAPS measurement.

### 2.3. Data Collection

Primary outcome measures are behavioral changes, including healthy eating, increased physical activity according to the application and questionnaire responses, and fitness measurements from the PAPS. Secondary outcome measures are changes in anthropometric parameters (body weight, height, BMI z-scores, obesity prevalence, and waist circumference) based on PAPS, BMI percentile, and psychological variables, such as self-body perception and sleep hours, reported in the questionnaires. Information from PAPS was measured at the time of enrollment, as well as at three months and at nine months following the trial.

### 2.4. Statistical Analysis

Data is analyzed using statistical software SPSS (version 21.0, IBM Corp., Armonk, NY, USA). Body mass index (BMI) is expressed in percentile according to Korean growth charts [33]. Comparisons of continuous variables between groups within sexes are performed using unpaired *t*-tests or Wilcoxon or Mann-Whitney tests as appropriate, after testing variables for normality of distributions and equality of variances. Comparisons of continuous variables after the trial within groups are performed using paired *t*-tests, Mann-Whitney U tests, or Wilcoxon signed ranks test as appropriate. Chi-square tests or Fisher’s exact probability tests are used to test differences between groups for categorical variables. Comparisons between groups of combined sexes are performed using linear (continuous outcomes) or logistic (categorical outcomes) regressions adjusted for sex, baseline BMI z-score, duration of application usage, and school.

### 2.5. Ethical Aspects of the Study

The study protocol, questionnaires, consent forms for both children and parents, and study plans were approved by the Inje University Paik Hospital’s Institutional Review Board (IIT-2015-070). This study is registered by CRIS (Clinical Research Information Service) with registration number KCT0002105.

## 3. Discussion

The purpose of this study is to evaluate the effectiveness of obesity prevention in children using a mobile platform with smartphones and wearable devices. Increasing physical activity and healthy eating is the best strategy for obesity prevention. The HAPPY ME platform was developed for the purpose of obesity prevention in children. We developed the platform with a comprehensive approach based on a multi-component intervention strategy following established recommendations.

There are some limitations that need to be addressed. First, the current trial was not randomized or blind controlled. School involvement is one of the most important aspects of the trial—as school motivates children—but schools are the most difficult group to recruit for randomized controlled trials (RCTs), especially when smartphones are involved. Schools do not allow random allocation. The participating schools herein agreed to join this project only once they were apprised of the allocation of students, as well as all process and content provided to the children. Most of the schools wanted to be involved in the intervention group. Blinding was also impossible for the intervention and control groups, which may have added bias to the results. During our forthcoming analysis, we will try to control related factors as confounders and effect modifiers. In addition, we will try to identify the difference between users and non-users following the trial, beyond only the intervention and control groups. Secondly, the duration of the trial is less than a year, whereas obesity prevention studies recommend longer durations [11,12]. Therefore, we could not use obesity prevalence or BMI z-scores as primary outcomes. Nevertheless, behavior changes are the most important outcomes in achieving a healthy weight in the long term. Thirdly, students who owned smartphones with Android operating systems were able to join the study in the intervention group. More than 90% of fifth-sixth graders possess smartphones, and more than 95% of them have Android systems. Finally, we could not develop gender-specific programs, even though it is known that boys tend to be more obese than girls [1,2,36]. Sugar intake also varies by sex [33]. Separate analysis in boys and girls would control those confounders, however, making clear which factors are more related to gender in childhood obesity. Future trials to develop and assess the effects of gender-specific programs for long-term prevention of childhood obesity, together with the integration of schools, are needed. Because there is evidence that low socioeconomic status and low fruit and vegetable intakes are associated with obesity, it would be more effective if future studies were able to target specific gender and socioeconomic samples for intervention [16,25]. The results of this study will offer evidence of the effectiveness of a multi-component prevention program that involves both schools and parents together with the use of smartphones and wearable devices.

## 4. Conclusions

The results of this study will offer evidence of the effectiveness of a mobile platform service with a multi-component intervention program based on a comprehensive approach.

## Figures and Tables

**Table 1:** Timeline of the study protocol procedure.

Study Procedure	Safety Test	Efficacy Test				Long-Term Effect Test
	A Week before	Baseline	Week 4	Week 8	Week 12	Month 6
1. Workshop for intervention group	I	I				
2. Screen-time monitoring	I	I	I	I	I	
3. Group education session		I, C		I, C		
4. Lifestyle questionnaire		I, C			I, C	I, C
5. PAPS measurement		I, C			I, C	I, C

I: Intervention group, C: Control group.

## References

[1] Oh K., Jang M.J., Lee N.Y., Moon J.S., Lee C.G., Yoo M.H., Kim Y.T. (2008). Prevalence and trends in obesity among Korean children and adolescents in 1997 and 2005. Korean J. Pediatr..

[2] OECD (2015). Overweight and obesity among children. Health at a Glance 2015 ed.

[3] Centers for Disease Control and Prevention. https://www.cdc.gov/obesity/childhood/defining.html.

[4] Ogden C.L., Yanovski S.Z., Carroll M.D., Flegal K.M. (2007). The epidemiology of obesity. Gastroenterology.

[5] Ebbeling C.B., Pawlak D.B., Ludwig D.S. (2002). Childhood obesity: Public-health crisis, common sense cure. Lancet.

[6] Waters E., de Silva-Sanigorski A., Burford B.J., Brown T., Campbell K.J., Summerbell C.D. (2011). Interventions for preventing obesity in children (Review). Cochrane Database Syst. Rev..

[7] Sisson S.B., Krampe M., Anundson K., Castle S. (2016). Obesity prevention and obesogenic behavior interventions in child care: A systematic review. Prev. Med..

[8] Benjamin Neelon S.E., Ostbye T., Hales D., Vaughn A., Ward D.S. (2016). Preventing childhood obesity in early care and education settings: Lessons from two intervention studies. Child Care Health Dev..

[9] Eldridge G., Paul L., Bailey S.J., Ashe C.B., Martz J., Lynch W. (2016). Effects of parent-only childhood obesity prevention programs on BMIz and body image in rural preteens. Body Image.

[10] Lazorick S., Fang X., Crawford Y. (2016). The MATCH Program: Long-Term Obesity Prevention through a Middle School Based Intervention. Child Obes..

[11] Granado-Font E., Flores-Mateo G., Sorli-Aguilar M., Montana-Carreras X., Ferre-Grau C., Barrera-Uriarte M.L., Oriol-Colominas E., Rey-Renones C., Caules I., Satue-Gracia E.M. (2015). Effectiveness of a Smartphone application and wearable device for weight loss in overweight or obese primary care patients: Protocol for a randomised controlled trial. BMC Public Health.

[12] Coughlin S.S., Whitehead M., Sheats J.Q., Mastromonico J., Smith S. (2016). A Review of Smartphone Applications for Promoting Physical Activity. Jacobs J. Community Med..

[13] Wang Y., Cai L., Wu Y., Wilson R.F., Weston C., Segal J. (2015). What Childhood Obesity Prevention Programs work? A systemic review and Meta-Aanalysis. Obes. Rev..

[14] Avis J.L., Cave A.L., Donaldson S., Ellendt C., Holt N.L., Jelinski S., Martz P., Maximova K., Padwal R., Wild T.C. (2015). Working With Parents to Prevent Childhood Obesity: Protocol for a Primary Care-Based eHealth Study. JMIR Res. Protoc..

[15] Smith J.J., Morgan P.J., Plotnikoff R.C., Dally K.A., Salmon J., Okely A.D., Finn T.L., Babic M.J., Skinner G., Lubans D.R. (2014). Rationale and study protocol for the “active teen leaders avoiding screen-time” (ATLAS) group randomized controlled trial: An obesity prevention intervention for adolescent boys from schools in low-income communities. Contemp. Clin. Trials.

[16] Shin D.W., Joh H.K., Yun J.M., Kwon H.T., Lee H., Min H., Shin J.H., Chung W.J., Park J.H., Cho B. (2016). Design and baseline characteristics of participants in the Enhancing Physical Activity and Reducing Obesity through Smartcare and Financial Incentives (EPAROSFI): A pilot randomized controlled trial. Contemp. Clin. Trials.

[17] Carriere C., Cabaussel C., Bader C., Barberger-Gateau P., Thilbault H. (2016). Multidisciplinary care management has a positive effect on pediatric obesity and social and individual factors are associated with better outcomes. Acta Paediatr..

[18] Amini M., Djazayery A., Taghdisi M.H., Nourmohammadi M. (2016). A school-based intervention to reduce excess weight in overweight and obese primary school students. Biol. Res. Nurs..

[19] Franklin C.G., Kim J.S., Montgomery K.L. (2012). Teacher involvement in school mental health interventions: A systematic review. Child Youth Serv. Rev..

[20] Jane M., Foster J., Hagger M., Pal S. (2015). Using new technologies to promote weight management: A randomised controlled trial study protocol. BMC Public Health.

[21] Park B.K., Nahm E.S., Rogers V.E., Choi M., Friedmann E., Wilson M., Koru G. (2017). A Facebook-Based Obesity Prevention Program for Korean American Adolescents: Usability Evaluation. J. Pediatr. Health Care.

[22] Wilkie H.J., Standage M., Gillison F.B., Cumming S.P., Katzmarzyk P.T. (2016). Multiple lifestyle behaviors and overweight and obesity among children aged 9–11 years: Results from the UK site of the International Study of Childhood Obesity, Lifestyle and the Environment. BMJ Open.

[23] Lee H.A., Park H. (2016). The mediation effect of individual eating behaviors on the relationship between socioeconomic status and dietary quality in children: The Korean National Health and Nutrition Examination Survey. Eur. J. Nutr..

[24] Neufel N.D. (2016). Outcome analysis of the B.E. S.T.R.O.N.G. Childhood obesity treatment program: Effectiveness of an eight-week family-based childhood obesity program using an internet-based health tracker. Child Obes..

[25] Rollo M.E., Aquiar E.J., Collins C.E. (2016). eHealth technologies to support nutrition and physical activity behaviors in diabetes self-management. Diabetes Metab. Syndr. Obes..

[26] Schoeppe S., Alley S., Van Lippevelde W., Vandelanotte C. (2016). Efficacy of interventions that use apps to improve diet, physical activity and sedentary behavior: A systematic review. Int. J. Behav. Nutr. Phys. Act..

[27] Fisher E.B., Fitzgibbon M.L., Glasgow R.E., Haire-Joshu D., Hayman L.L., Kaplan R.M., Nanney M.S., Ockene J.K. (2011). Behavior matters. Am. J. Prev. Med..

[28] Tate E.B., Spruijt-Metz D., O’Reilly G., Jordan-Marsh M., Gotsis M., Dunton G.F. (2013). mHealth approaches to child obesity prevention: Successes, unique challenges, and next directions. TBM.

[29] Jensen C.D., Duncombe K.M., Woolford S.J. (2016). An Evaluation of a smartphone-assisted behavioral weight control intervention for adolescents: Pilot study. JMIR Mhealth Uhealth.

[30] Quelly S.B., Norris A.E., DiPietro J.L. (2016). Impact of mobile apps to combat obesity in children and adolescents: A systematic literature review. J. Spec. Pediatr. Nur..

[31] Kim Y.H. (2015). Analysis in cell phone use in children and adolescents. KISDI Stat. Rep..

[32] Kim H., Kang J.-H., Park H.A., Cho S.H., Jeon S., Jung J.-H., Sung E. (2015). Development of a Smartphone Application Prototype for Child Obesity Prevention: Rationale and Study Design of Acceptability and Feasibility Tests. Korean J. Health Promot..

[33] Korea Centers for Disease Control and Prevention. http://cdc.go.kr/CDC/notice/CdcKrInfo0201.jsp?menuIds=HOME001-MNU1154-MNU0005-MNU1889&cid=1235.

[34] Ha K., Chung S., Lee H.S., Kim C.I., Joung H., Paik H.Y., Song Y. (2016). Association of Dietary Sugars and Sugar-Sweetened Beverage Intake with Obesity in Korean Children and Adolescents. Nutrients.

[35] Lee S.K., Kim M.K. (2016). Relationship of sodium intake with obesity among Korean children and adolescents: Korea National Health and Nutrition Examination Survey. Br. J. Nutr..

[36] Kim S.H., Song Y.H., Park S., Park M.J. (2016). Impact of lifestyle factors on trends in lipid profiles among Korean adolescents: The Korea National Health and Nutrition Examination Surveys study, 1998 and 2010. Korean J. Pediatr..

[37] Finch M., Jones J., Yoong S., Wiggers J., Wolfenden L. (2016). Effectiveness of centre-based childcare interventions in increasing child physical activity: A systematic review and meta-analysis for policymakers and practitioners. Obes. Rev..

[38] O’Malley G., Clarke M., Burls A., Murphy S., Murphy N., Perry I.J. (2014). A smartphone intervention for adolescent obesity: Study protocol for a randomised controlled non-inferiority trial. Trials.

[39] Jung J.-H., Jeon S.H., Bae H.J., Cho Y.-G., Hur Y.-I., Sung E.J., Kang J.-H. (2016). Development of a Smartphone Application for 4th–6th Grade Elementary Students Aimed to Prevent Childhood Obesity. Korean J. Obes..

[40] Tudor-Locke C., Craig C.L., Beets M.W., Belton S., Cardon G.M., Duncan S., Hatano Y., Lubans D.R., Olds T.S., Raustorp A. (2011). How many steps/day are enough? For children and adolescents. Int. J. Behav. Nutr. Phys. Act..

[41] Green Cross Health Care (2014). Report on the Accuracy of an Exercise Index of an Activity Tracker (Report).

